# Aging-Related Alterations to Persistent Firing in the Lateral Entorhinal Cortex Contribute to Deficits in Temporal Associative Memory

**DOI:** 10.3389/fnagi.2022.838513

**Published:** 2022-03-11

**Authors:** Carmen Lin, M. Matthew Oh, John F. Disterhoft

**Affiliations:** Department of Neuroscience, Feinberg School of Medicine, Northwestern University, Chicago, IL, United States

**Keywords:** excitability, prefrontal, hippocampus, acetylcholine, afterhyperpolarization (AHP), afterdepolarization

## Abstract

With aging comes a myriad of different disorders, and cognitive decline is one of them. Studies have consistently shown a decline amongst aged subjects in their ability to acquire and maintain temporal associative memory. Defined as the memory of the association between two objects that are separated in time, temporal associative memory is dependent on neocortical structures such as the prefrontal cortex and temporal lobe structures. For this memory to be acquired, a mental trace of the first stimulus is necessary to bridge the temporal gap so the two stimuli can be properly associated. Persistent firing, the ability of the neuron to continue to fire action potentials even after the termination of a triggering stimulus, is one mechanism that is posited to support this mental trace. A recent study demonstrated a decline in persistent firing ability in pyramidal neurons of layer III of the lateral entorhinal cortex with aging, contributing to learning impairments in temporal associative memory acquisition. In this work, we explore the potential ways persistent firing in lateral entorhinal cortex (LEC) III supports temporal associative memory, and how aging may disrupt this mechanism within the temporal lobe system, resulting in impairment in this crucial behavior.

## Introduction

Few things are as widely associated with aging as cognitive decline. Compared to young controls, aged subjects have significant defects in working memory, attention, task-switching, executive function, and declarative memory (Grady, 2012).

Decline in performance on temporal associative memory tasks, in particular, has been extensively studied in aging research. Associative memory is the memory of the relationship between two objects and can be either declarative or non-declarative. The presence of a stimulus free temporal interval between the two objects makes the memory declarative, and dependent on temporal lobe structures such as the hippocampus and entorhinal cortices.

A paradigm of temporal associative memory that is often used is trace eyeblink conditioning (tEBC). In tEBC, a neutral conditioned stimulus (CS; typically a tone or flash of light) is paired with a biologically salient unconditioned stimulus (US; airpuff to the eye or electrical shock to the periorbital area) that elicits a reflexive eyeblink response known as the unconditioned response (UR). The CS and the US are separated by a temporal interval of a few hundred milliseconds to seconds (depending on the species of the subject) to make the paradigm temporal-lobe dependent. After multiple pairings of the CS and the US, the CS, which had previously been neutral is able to elicit an eyeblink response that precedes the onset of the US. This learned eyeblink response is known as the conditioned response (CR) (Disterhoft and Oh, 2006; Clark, 2011).

Aging-related deficits in tEBC has been observed across many mammalian species, including mice, rats, rabbits, and humans (Thompson et al., 1996a; Knuttinen et al., 2001a,b; Disterhoft and Oh, 2006; Galvez et al., 2011; Kennard and Woodruff-Pak, 2011; Lin et al., 2020). There are multiple structural and functional neural changes that underlie the performance deficits in temporal associative tasks such as tEBC. This review examines how changes in persistent firing in the lateral entorhinal cortex (LEC) contributes to alterations in the entorhinal-hippocampal circuitry that supports this behavioral paradigm, leading to impairments.

## Persistent Firing in Temporal Lobe Structures

**FIGURE 1:**
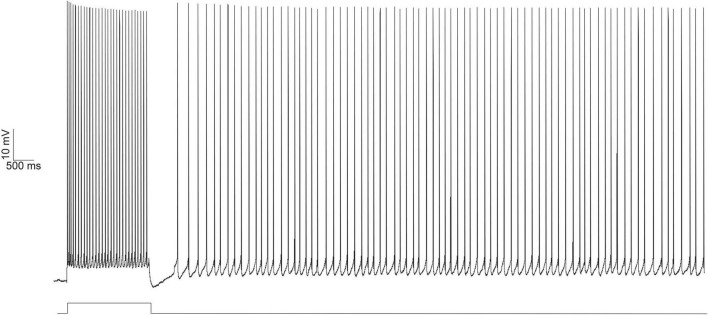
Persistent firing example trace from an LEC neuron evoked with depolarizing current injection using *in vitro* electrophysiology. Persistent firing activity of the neuron evoked with whole cell current clamp (top trace). A 100 pA, 2 s long depolarizing current (bottom trace, protocol as reported in Tahvildari et al., 2008) was injected into the neuron, eliciting an initial 2 s burst of action potentials. Even after the termination of the depolarizing current injection, the neuron continues to fire action potentials. From Lin (2020).

Persistent firing is the ability of a neuron to continue firing action potentials, even after the termination of a triggering stimulus (Figure 1). It was first discovered in the prefrontal cortex of monkey subjects as they performed a working memory task. During the task, monkeys needed to remember the location of a food reward (cue) so it could access the reward following a stimulus-free delay period. Single neuron recordings from the prefrontal cortex showed that neurons became active during the initial viewing of the reward, and maintained their activity throughout the delay period, terminating their activity when the monkey had accessed the reward. In trials when no reward was presented, the neurons that had been active when the reward was present were quiescent (Fuster, 1972). The persistent activity displayed by prefrontal neurons during the delay period is thought to support a neural trace of the location of the reward, allowing the monkey to access it at a later timepoint. Inhibition of prefrontal persistent activity during a working memory task has been shown to significantly impair performance on such a task (Liu et al., 2014). As a result, persistent firing is generally thought to support memory-guided movement planning.

Since the seminal study by Fuster (1972), persistent firing has been found in multiple areas of the brain, including medial temporal lobe structures such as the hippocampus, perirhinal, and entorhinal cortices (Egorov et al., 2002; Tahvildari et al., 2007, 2008; Yoshida et al., 2008; Navaroli et al., 2012; Jochems and Yoshida, 2013; Knauer et al., 2013; Zylberberg and Stowbridge, 2017). These regions exhibit persistent firing during working and temporal associative memory tasks. One human single neuron recording study found increased hippocampal neuronal firing rates 500–1,500 ms after the cue, extending into the delay period of the task (Staresina et al., 2019). Studies in non-human primates also found increased firing activity in the entorhinal cortex (EC) during the delay period of delayed match-to-sample and delayed non-match-to-sample tasks (Suzuki et al., 1997; Young et al., 1997).

In both the lateral and medial entorhinal (MEC) cortices, persistent firing has been found in the neurons of layer III (Tahvildari et al., 2007, 2008; Yoshida et al., 2008). One study from our laboratory found a CS-evoked increase in activity from the superficial layers (i.e., layers II and III) of the LEC *in vivo* that continued into the trace interval during acquisition of tEBC (Suter et al., 2019). Blocking layer III input from the MEC has also been shown to impair acquisition of a temporal associative paradigm (Suh et al., 2011).

Evoking persistent firing in entorhinal regions *in vitro* requires suprathreshold stimulation of the neuron in the presence of the muscarinic agonist, carbachol. The suprathreshold stimulation evokes a train of action potentials, but the neuron continues firing action potentials even after stimulus termination (Figure 1). Persistent firing activity is only terminated as a result of external influence (e.g., a second depolarizing stimulus) (Tahvildari et al., 2007, 2008). For memory researchers, the initial suprathreshold stimulation that evokes firing activity could come from the CS in a tEBC paradigm and persistent firing is a way for behaving animals to hold the CS in its working memory during the stimulus-free trace interval.

Despite several studies implicating the contribution of persistent firing to temporal associative learning, none have directly investigated the role persistent firing plays in this paradigm. Thus, Lin et al. (2020) addressed this gap in knowledge by investigating whether and how learning changes persistent firing in LEC III principal neurons *in vitro*. They trained young adult rats on tEBC and found an increase in persistent firing ability (measured as increased persistent firing probability and firing rate) in LEC III neurons compared to behavioral controls.

**FIGURE 2:**
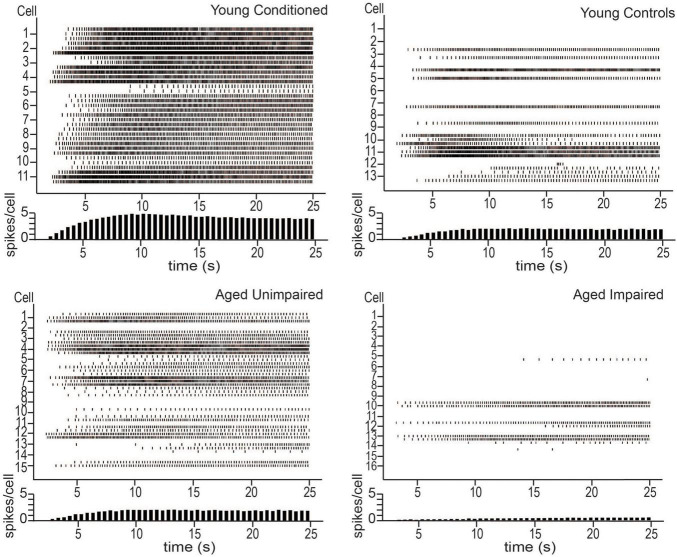
Persistent firing activity from young animals trained on tEBC **(top left)**, young behavioral controls **(top right)**, aged unimpaired **(bottom left)**, and aged impaired **(bottom right)** animals. Persistent firing activity evoked using *in vitro* intracellular current injection. Three sweeps were evoked for each cell. Each row represents one sweep of activity, with each cell marked by its second sweep of activity. Below each plot is a histogram of average spikes/cell across the sweep. Young animals trained on tEBC have more persistent activity compared to behavioral controls. Aged impaired animals are relatively inactive. Reproduced from Lin et al. (2020).

Lin et al. (2020) also examined persistent firing from behaviorally naïve and conditioned aged animals *in vitro*. There was a decrease in persistent firing ability in aged naïve animals compared to young controls, indicating that normal aging alters persistent firing ability in LEC III. When aged animals were trained on the trace eyeblink task, the animals separated naturally into those that were able to form the association between the CS and the US (learning unimpaired), and those that were not (learning impaired), as has been reported in previous aging studies (Thompson et al., 1996a; Knuttinen et al., 2001a; Tombaugh et al., 2005; Disterhoft and Oh, 2006). The unimpaired animals were able to achieve the same behavioral performance as young animals who were trained on the task, and they had increased persistent firing ability compared to learning impaired animals. However, neurons from aged unimpaired animals still had a lower persistent firing capacity compared to young animals who learned the task (Figure 2).

Functional *in vivo* recordings are still required to verify the role of persistent firing in supporting a task such as tEBC, however, the results of Lin et al. (2020) imply a potential synergy between learning and persistent firing in LEC III. The results suggest that persistent firing is important for successful acquisition of tEBC and that successful acquisition enhances persistent firing ability. Aged animals, especially those that are learning impaired, have limited persistent firing ability. This implies aging causes animals to lose their persistent firing ability in brain regions important for associative learning, such as the LEC, contributing to the learning impairments that are often observed in the aged population.

## Role of Persistent Firing in Supporting Temporal Associative Learning

To understand how aging-related alterations in persistent firing leads to impairments in temporal associative learning, we must first understand how persistent firing supports acquisition of such a task. While there has been considerable speculation on this topic (i.e., timing of persistent firing or the information that it encodes), there has so far not been sufficient evidence to define its role, nor how it fits into the overall functional circuitry in the temporal lobe.

However, we can begin to form an idea based on the evidence of previous studies investigating persistent firing, hippocampal-entorhinal physiology and circuitry, and learning-induced functional changes in the neurons of these regions. This section will lay out three theories on how persistent firing in LEC III may support temporal associative learning. While persistent firing may support any task that requires the association between temporally discontinuous stimuli, the theories laid out below will use tEBC as an example.

### Persistent Firing Bridges the Trace Interval in Trace Eyeblink Conditioning

**FIGURE 3:**
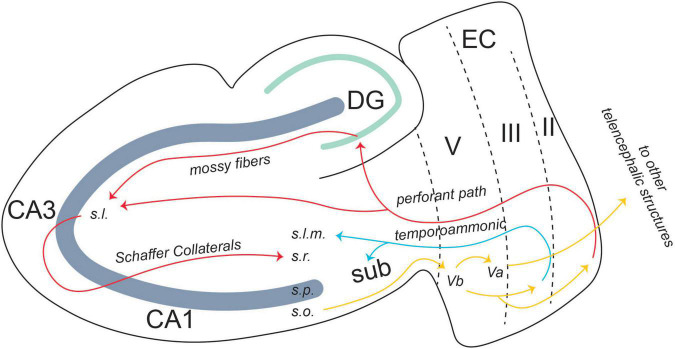
Entorhinal-hippocampal circuitry. There are two pathways of information flow from the EC to the CA1 region: the temporoammonic pathway (originating in layer III) and the perforant path (originating in layer II). From the CA1 region, the information flows back to layer V, where it is projected to other telencephalic structures or back to layers II and III in an entorhinal-hippocampal loop. From Lin (2020).

The EC, both lateral and medial, is the main relay station for information moving into and out of the hippocampus. Within an intact medial temporal lobe system, the perirhinal and parahippocampal cortices receive information processed by unimodal and multimodal cortical regions and send the information to the EC. There are two pathways of information flow to the CA1 region of the hippocampus from the EC. The first is the direct or temporoammonic pathway (TA) (van Groen et al., 2003; Squire et al., 2004). This pathway originates in layer III of the EC and projects directly to CA1. The other pathway for information flow is the canonical hippocampus trisynaptic circuit (Figure 3).

There is also an output pathway from the CA1 region back to the EC. Layer V of EC is split into two separate layers: Va and Vb. CA1 projects back to Vb of the EC. Vb itself has two separate projections. One is to Va, where it feeds back out to other telencephalic structures such as the nucleus accumbens, the amygdala, and the medial prefrontal cortex (mPFC) (Figure 3; Ohara et al., 2018). The other projection from Vb is back to layers II and III of the EC. The connection between layer Vb and II/III allows for information from the hippocampus to be fed back into itself, forming a hippocampal-entorhinal loop. Both these projections are thought to promote the consolidation of the memory into long-term storage (Ohara et al., 2018).

As mentioned above, the general theory behind persistent firing’s role in a working memory task is that it supports the mental trace of a cue during the delay period (Kitamura et al., 2015; Zylberberg and Stowbridge, 2017; Constantinidis et al., 2018; Lundqvist et al., 2018). Using the example of tEBC, persistent firing will maintain the information about the CS during the trace interval so the stimulus can be properly associated with the US. Since layer III of the EC projects directly to CA1, a region critical for the temporal binding of the CS and the US, persistent firing in LEC III is a prime candidate for maintaining the information about the CS during the trace period.

Several pieces of evidence exist to support this theory. First, the LEC is crucial for the acquisition of associative and temporal associative tasks. Inactivation or lesions to this region have been shown to result in impairments in these tasks, but not in control non-associative novel object recognition tasks (Morrissey et al., 2012; Wilson et al., 2013a). Second, evidence suggests that the LEC provides information on salient stimuli to the hippocampus, either about the stimuli themselves through salience-gated object processing or by simply firing when the subject attends to a salient stimulus (Deshmukh and Knierim, 2011; Wilson et al., 2013b). This indicates that the LEC can support incoming information about the CS and the US. Finally, the LEC is able to support the precise timing and sequencing of events (Bellmund et al., 2019; Montchal et al., 2019). Within the LEC, there exist time cells, which can encode time across an entire behavioral task as well as within a single trial (Tsao et al., 2018). This ability of the LEC to build an accurate temporal representation of events could support the mental formation of the temporal relationship between the CS and US in trace conditioning, and persistent firing may be a mechanism by which that temporal relationship can be formed.

Persistent firing in LEC III requires cholinergic innervation. Studies that evoke persistent firing *in vitro* require the application of the muscarinic agonist, carbachol (Tahvildari et al., 2007, 2008; Esclassan et al., 2009). The EC is heavily innervated by cholinergic projections from the medial septum and the vertical limb of the diagonal band of Broca of the basal forebrain (Insausti et al., 1997; Heys et al., 2012). In addition, cholinergic tone has been shown to increase in temporal lobe regions such as the CA1 region during tEBC (Oh et al., 1999; Disterhoft and Oh, 2003). Since acetylcholine is important in attention and attending to salient environmental factors, the US, a salient stimulus, may recruit acetylcholine (Picciotto et al., 2012). Cholinergic innervation activates an intrinsic membrane bistability (Up and Down states) in LEC neurons. CS information is then able to activate sustained activity (Up state) (Heys et al., 2012).

**FIGURE 4:**
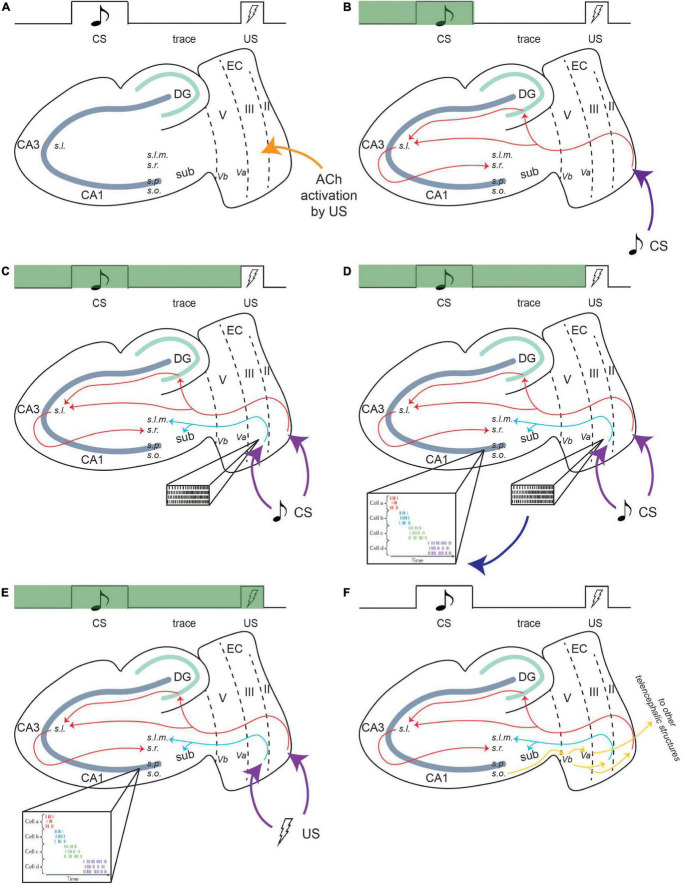
Persistent firing bridges the trace period in tEBC. Green bar indicates progress during a single trial of tEBC. In this example, the CS is an auditory tone and the US is a mild electrical shock to the periorbital area. Due to its saliency, the US activates cholinergic innervation to the LEC region **(A)**. Then, CS information projects to the CA1 region of the hippocampus *via* the PF path **(B)**. For the mental trace of the CS to be maintained, the CS also activates persistent firing in LEC III. LEC III projects directly to CA1 *via* the TA pathway **(C)**, which puts it in a prime position to stimulate and sustain CA1 activity throughout the trace interval. LEC III persistent firing may activate time cells in the CA1 region, which develops firing patterns time-locked to sequential moments of the trial **(D)**, time cell activity adapted from Eichenbaum (2014). The activity of time cells bridges the trace interval and can maintain the CS information during the interval until US information arrives **(E)**. Following the acquisition of tEBC, the learned behavior is projected *via* output pathways to be consolidated into long-term storage **(F)**. From Lin (2020).

Layers II and III of the LEC receive CS and US information from the perirhinal and parahippocampal cortices and relay it to the CA1 region, which is essential for the formation of the temporal relationship (Figure 4; Potier et al., 2016; Sellami et al., 2017; Al Abed et al., 2020). Optogenetic inhibition of this area results in impairment in recall of the conditioning paradigm (Sellami et al., 2017). LEC II relays CS information to the CA1 region of the hippocampus *via* the PF path (Figure 4). Due to its projection to CA1 *via* the TA pathway, persistent firing in LEC III may sustain CA1 activity during the trace interval, eliciting persistent firing within CA1 neurons, thus allowing for CS information to be maintained until the US information arrives *via* the trisynaptic pathway originating in layer II (Solomon et al., 1986; Knauer et al., 2013).

Time cell activity in the CA1 region may assist in the formation of the CS–US relationship. The function of time cells in CA1 is slightly different from time cells in the LEC. In a temporally structured event or memory, time cells in the hippocampus fire at specific epochs. Ensembles of time cell activity, together, can encode an entire temporal period (Eichenbaum, 2014). Time cells have been found to be active during tEBC, with their firing sequences time-locking to specific moments in a single trial. During tEBC, the CS, the trace period, and US are eventually encoded by the firing sequence of an ensemble of time cells (Eichenbaum, 2014; Modi et al., 2014). Time cells may also be able to actively maintain the CS information. In a study which utilized a working memory task involving two distinct cues, each paired with its own target, researchers found that the time cells that were active during the delay period altered their firing pattern depending on the identity of the cue (MacDonald et al., 2013). This indicates that time cell activity contains information about the specific event, perhaps to inform future behavior.

It has also been suggested that entorhinal input that bridges the temporal gap between associated events could help the timing evolve for time cells (MacDonald et al., 2011). As such, persistent firing in LEC III could drive CA1 time cell activity during tEBC acquisition. Time cells would encode the precise temporal sequence of a single trial and maintain CS information over the trace interval (Figure 4).

### Persistent Firing Enhances Schaffer Collateral-Evoked Activity to CA1

While the primary theory for persistent firing in LEC III is that it bridges the trace interval during tEBC, this theory has not yet been confirmed using functional recordings. Persistent firing in LEC III may support the acquisition of temporal associative tasks *via* other ways. Another possible role for persistent firing is to enhance Schaffer Collateral (SC) input to its direct downstream target, CA1 pyramidal neurons.

Temporoammonic activation from LEC III to CA1 can modulate SC-evoked CA1 activity (Remondes and Schuman, 2002). A burst of TA activity that immediately precedes a previously ineffective SC stimulation can facilitate a SC-evoked spike. Furthermore, plasticity (i.e., potentiation or depression) at the TA-CA1 synapse is able to modulate (i.e., enhance or inhibit) the spike enhancing ability of the TA pathway (Remondes and Schuman, 2002).

**FIGURE 5:**
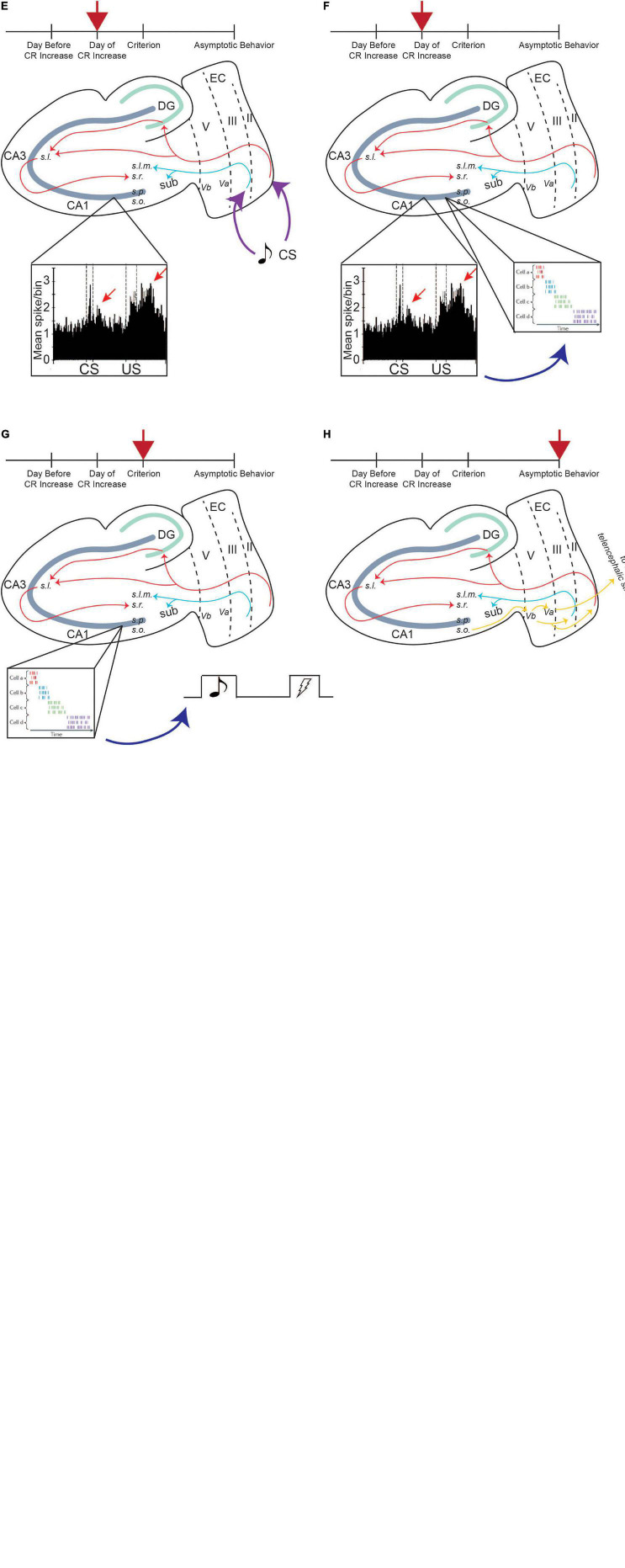
Persistent firing enhances input to CA1 pyramidal neurons, allowing for successful learning. Red arrow indicates timepoint in training. The day before CR increase, CA1 encodes the saliency of US, and activity increases in response to US **(A)**, inset, enhanced CA1 activity to US presentation, adapted from McEchron and Disterhoft (1999). Enhanced CA1 activity feeds back to LEC III and decreases the AHP, increasing the excitability of these neurons **(B)**, which allows it to persistently fire more easily. Incoming CS information can activate persistent firing in LEC III **(C)**, inset, persistent firing). Persistent firing can enhance SC input containing CS information, which is being projected to the CA1 region *via* the PF path **(D)**, inset, TA firing enhances SC input to CA1 neurons, adapted from Remondes and Schuman (2002). CA1 neurons increase their activity in response to CS presentation **(E)**, inset, enhanced CA1 activity to CS and US presentation, adapted from McEchron and Disterhoft (1999). Enhanced CA1 activity to the stimuli can drive sequential time-locking in time cells **(F)**, inset, time cell activity, adapted from Eichenbaum (2014). Time-locking encodes CS, trace interval, and US sequence within a single trial, helping the animal bridge the temporal gap between the stimuli, and is stabilized when the animal reaches a criterion for learning (i.e., when the animal exhibits consistent CRs) **(G)**. Once the animal learns the paradigm well and reaches asymptotic behavior, the behavior is moved out of the hippocampus *via* output pathways to be consolidated into long-term memory **(H)**. From Lin (2020).

Persistent firing in LEC III may provide the TA pathway modulation of SC input to CA1 neurons. This could allow for the CA1 region to be more responsive to convergent CS and US information provided *via* the tri-synaptic pathway (Figure 5). CA1 activity has been shown to increase in response to the CS and the US during acquisition of tEBC (McEchron and Disterhoft, 1997, 1999; McEchron et al., 2001). *In vitro* recordings have also seen enhanced synaptic transmission (measured as larger EPSPs in CA1 neurons) between the CA3 and CA1 neurons following successful tEBC acquisition, supporting the idea that enhanced SC input to CA1 underlies successful acquisition (Power et al., 1997; Gruart et al., 2006).

Activity change within CA1 neurons occurs over multiple sessions of tEBC (Figure 5). These neurons increase their activity first to the US, which is evident the day before the behavioral expression of learning (i.e., before a significant increase in the number of CRs the animal exhibits). This may be because the hippocampus first needs to encode the saliency of the US, which, again, may require acetylcholine recruitment (Picciotto et al., 2012). Enhanced CA1 activity in response to the US would then feed back into layer III of the LEC. Within LEC III neurons, synaptic excitability changes could enhance the intrinsic excitability of LEC III pyramidal neurons, decreasing the size of the postburst afterhyperpolarization (AHP), a modulator of intrinsic excitability. Cholinergic recruitment by the US to these temporal lobe regions will also place LEC III neurons in a bistable state, facilitating persistent firing activation by incoming CS information.

Persistent firing-induced TA activity could then enhance SC input to CA1 containing information about the more neutral CS, allowing CA1 neurons to fire in response to CS presentation. Enhanced CA1 activity in response to both the CS and the US may help CA1 time cells to time-lock their firing to sequential moments of an individual trial of tEBC. The time-locking function of time cells emerges progressively over the course of conditioning, with the time-locked sequences established by the time the animal has learned the paradigm (Modi et al., 2014). Activity from time cells could bridge the trace interval between the CS and the US, sustaining the CS information, and allowing for the association between the two stimuli to be formed (MacDonald et al., 2013; Modi et al., 2014). Once that association is formed, the animal would start exhibiting CRs and the learned behavior can be consolidated into long-term memory (Figure 5).

Thus far, we have discussed two possible roles for LEC III persistent firing in supporting tEBC. It is necessary to point out that these two roles are not necessarily mutually exclusive of each other. Functionally, a combination of the two may exist. LEC III persistent firing may serve to enhance SC input containing stimulus information to CA1 neurons while also supporting the formation of time cell activity, which could sustain CS information and bridge the trace interval. In addition, there are two important aspects related to persistent firing in LEC III that we should address here. The first is to do with the length of persistent ability with regards to the temporal length of the trace interval in tEBC. The second is to do with debate about the necessity of persistent firing in supporting working memory.

First, we will address the relationship between the length of the trace interval in tEBC and the length of persistence of LEC III persistent firing. In tEBC, an effective trace interval is at most a few seconds (in humans) and several hundred milliseconds in other species (i.e., 500 ms in rats). However, LEC III neurons can fire persistently and steadily for more than 25 s *in vitro/ex vivo*. The ability of these LEC neurons to fire for an extended period of time suggests that persistent firing may be more suited to trace fear rather than tEBC.

Indeed, recruitment of persistent firing is not exclusive to tEBC and may in fact support trace fear conditioning. However, the question remains: Why is the trace interval no longer than a few seconds for tEBC, if LEC III neurons can fire persistently for an extended period? Part of the reason probably comes from the fact that these paradigms recruit an extensive network of brain regions (e.g., brainstem/cerebellar circuitry for tEBC and amygdala for trace fear conditioning) (Schafe et al., 2005; Weiss and Disterhoft, 2011). These different brain regions may constrain the length of the trace interval for these paradigms. Another important portion of the answer could come from the level of cholinergic recruitment during trace fear and eyeblink paradigms.

While the amount of cholinergic activation elicited (by application of the muscarinic agonist carbachol) *in vitro* allows for LEC III neurons to sustain persistent firing activity for more than 25 s, it is possible that the level of cholinergic recruitment during tEBC is different *in vivo*. As we have established above, salient stimuli such as an electrical shock (as during a US) will recruit cholinergic activation. Guo et al. (2019) found activation of cholinergic basal forebrain neurons emerged over the course of training to mediate cortical responses to the CS and US during trace fear conditioning. Of note, basal forebrain neurons increased their activity during CS presentation and filled in the gap between the CS and US. This cholinergic activation modulated the excitability of pyramidal neurons in the primary sensory cortices (i.e., auditory cortex A1 for a tone CS) to promote representation of the auditory tone. Guo et al. (2019) went further to show that the timing of cholinergic modulation was highly malleable. An earlier study by Zhang et al. (1997) showed that acetylcholine concentration was a factor in the timing of this modulation. Application of acetylcholinesterase inhibitors lengthened the time constant by which acetylcholine could modulate neuronal excitability. Thus, we propose that the level of cholinergic recruitment determines the length of the trace interval, which is itself determined by the saliency of the US during trace fear and eyeblink conditioning. In other words, a more aversive US (as in trace fear conditioning) would recruit extended cholinergic activation relative to a less aversive US (as in tEBC). The extra activation could allow for an extended trace interval in trace fear conditioning. Ultimately, the length of LEC III persistent firing *in vivo* and the length of the trace interval would depend on the aversiveness of the US in a conditioning paradigm.

In addition, although LEC III neurons fire persistently and steadily for an extended period *in vitro*, it is not necessarily the case that this maintained activity occurs *in vivo*. Indeed, there is significant debate about the activity that occurs during the trace interval (Constantinidis et al., 2018; Lundqvist et al., 2018). Some argue that the maintained spiking observed (e.g., in Fuster, 1972; Goldman-Rakic, 1995) is the result of sparse activity that is averaged across trials. Some models of working memory posit that local networks of neural activity coordinate their activity during the delay period, and the combined activity of a population of neurons results in the persistent spiking activity observed. If this is true, the capability of LEC III neurons to fire as observed *in vitro* (i.e., sustained firing activity for an extended period of time) could allow for the development and evolution of a neural code *in vivo* that helps to maintain CS information (i.e., through modulation of firing frequency and timing) and so it can be incorporated into a neural network of activity to support the delay period (Lundqvist et al., 2018; Miller et al., 2018).

Regardless of whether LEC III persistent firing supports the CS by spanning the entire trace interval or allows the neurons to participate in an ensemble of activity that allows the trace interval to be bridged, LEC III persistent firing would be able to participate in the entorhinal-hippocampal functional circuitry we have illustrated above. In addition, it would certainly be able to support the final hypothesis for persistent firing, which is to synchronize the LEC with hippocampus and prefrontal cortical rhythms.

### Persistent Firing Allows for the Lateral Entorhinal Cortex to Couple With Hippocampal and Prefrontal Activity

The final role we will discuss for persistent firing in the LEC is its potential to allow the LEC to couple with hippocampal and prefrontal cortical rhythms. As mentioned previously, the LEC serves as an interface for information flowing between the hippocampus and cortical regions, and it has been suggested to play a similar role for the mPFC (Takehara-Nishiuchi, 2014). There are connections between the LEC and the mPFC that closely mirror those between the LEC and the hippocampus – i.e., the superficial layers (layers II and III) of the LEC project to the mPFC and receive projections from the mPFC to the deep layers (layer V) of the LEC (Insausti et al., 1997; Canto et al., 2008; Takehara-Nishiuchi, 2014).

Persistent firing in LEC could support the projection of cortically processed information to the mPFC during memory consolidation and expression, as it is proposed to do for the hippocampus during memory acquisition. During acquisition, the hippocampus integrates sensory information (e.g., auditory CS) from a large, distributed network of sensory and association cortical regions. Once the memory has been acquired, the memory becomes consolidated over a course of 2–4 weeks. During consolidation, the memory becomes independent of the hippocampus and the mPFC takes on the role of mediating the various cortical regions for consolidation and subsequent expression of the long-term memory (Scoville and Milner, 1957; Kim and Fanselow, 1992; Kim et al., 1995; Takehara et al., 2002, 2003; Frankland and Bontempi, 2005; Takehara-Nishiuchi and McNaughton, 2008).

Evidence from previous studies implicate the LEC in the consolidation of hippocampus-dependent memory. In one study, inactivating the LEC with muscimol, a GABA agonist, impaired the retrieval of not only a recently acquired (i.e., still hippocampus dependent), but also a long-term (i.e., hippocampus independent) memory of tEBC (Morrissey et al., 2012). In another study, lesions to the TA pathway impaired the memory of a spatial Morris Water Maze task when the animals were tested four weeks after initial acquisition (Remondes and Schuman, 2004).

During tEBC, enhanced synchronization of oscillations in the theta range (4–12 Hz) between the LEC, mPFC, and the hippocampus has been found (Takehara-Nishiuchi et al., 2011). Because persistent firing in LEC III is in the theta range (4–12 Hz), this mechanism may support the coupling of the LEC with the hippocampus during acquisition and with the mPFC during memory consolidation and expression (Lin et al., 2020).

During the initial acquisition of tEBC, synchronization between the hippocampus and LEC occurs following the cue or CS onset. This could support the transfer to and maintenance of CS information in the hippocampus (Takehara-Nishiuchi et al., 2011). Once the task has been successfully acquired (the animals’ performance reaches an asymptotic level), the synchronization declines between the LEC and the CA1 region, which may be the result of strengthening connections between the LEC and the mPFC.

Between the LEC and mPFC, synchronization is also strongest following CS onset, although the strength of the synchronization varies depending upon the type of trial and the timepoint during acquisition. During early acquisition, the LEC-mPFC synchronization is stronger for trials in which the animal did not exhibit a CR. The synchronization may drive mPFC neurons to encode the behavioral relevance of the CS, after first encoding US saliency (Takehara-Nishiuchi, 2020). However, later in acquisition, when performance is improving, the LEC-mPFC synchronization is stronger for trials in which the animal exhibits a CR. One month after acquisition, the synchronization is still strong, meaning that the connection between these two areas is important for expression of the consolidated memory (Takehara-Nishiuchi et al., 2011). Within prefrontal neurons, persistent firing activity has been found during recall of a consolidated but not recently acquired tEBC memory (Hattori et al., 2014). The activity started with the CS presentation and extended into the trace period, indicating that the role of mediating the CS-US association was transferred to prefrontal neurons as the memory became consolidated and the LEC-mPFC connection became responsible for memory retrieval.

**FIGURE 6:**
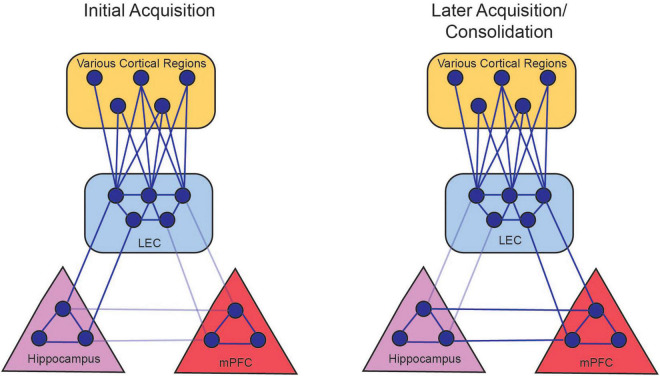
Persistent firing allows for the LEC to couple with hippocampal and prefrontal activity. During the initial acquisition of tEBC (left panel), persistent firing in LEC III allows for the LEC to synchronize with hippocampus oscillations in the theta range (solid lines). This allows the hippocampus to coordinate processed information from various cortical regions. Persistent firing also allows the LEC to couple with the mPFC, although this connection is not stabilized (faded lines) before the animal starts exhibiting increased CRs. During late acquisition and consolidation (right panel), LEC-hippocampus activation allows the LEC to strengthen connections with the mPFC, so the mPFC can take over the role of integrating the various cortical nodes that represents the memory. The LEC-hippocampus connection decreases in late acquisition (faded lines) as the LEC-mPFC connection strengthens (solid lines). There is also a projection from the hippocampus to the mPFC that can also support the consolidation of the memory (Takehara-Nishiuchi, 2014). From Lin (2020).

Thus, the initial and temporary LEC-hippocampus synchronization could allow the formation of the CS-US association that is necessary for the appropriate behavior to arise. The LEC-mPFC synchronization could reflect the mPFC taking over the role of mediating the CS-US association as it is consolidated into long-term memory, as well as the subsequent expression of memory (Figure 6).

## Neural Compensation in Aging

There are many outstanding reviews regarding cognitive maintenance, reserve, compensation, and resilience (e.g., Cabeza et al., 2018; Stern et al., 2020; McQuail et al., 2021). Therefore, we will focus our discussion to the potential mechanisms in the LEC that allow successful learning to occur in aged subjects.

With the essential role that persistent firing in LEC III seems to play in tEBC acquisition, it is perhaps surprising to see that aged learning unimpaired animals have comparable behavioral performance to young animals, despite their decreased persistent firing capacity (Lin et al., 2020). However, previous studies have indicated that the unimpaired aging brain is not simply maintenance of the younger brain, but a reorganization of neural networks to compensate for aging-related compromise. One study measuring metabolic activity with PET in young adult and aged human brains while subjects performed a working memory task showed that aged brains recruited a more anterior hippocampal network compared to young (i.e., dorsolateral prefrontal cortex vs. prefrontal cortex; middle cingulate gyrus vs. posterior cingulate). The aging brain, then, employs different neural circuits to achieve the same behavioral performance as young brains (Della-Maggiore et al., 2000; McKinney and Jacksonville, 2005).

Other studies have shown an increase in bilateral hemispheric communication in the aging brain that allows for young-like performance. During a memory recall task, prefrontal activation was more asymmetric in younger adults (with higher activation in right prefrontal areas). Older adults with comparable memory performance showed more bilateral prefrontal activation, while older adults with lower performance maintained asymmetric activation (Cabeza et al., 2002). To support the idea that cognitive reserve in older adults is dependent on an ability to recruit a more distributed neural network, Cabeza et al. (2002) applied a low frequency stimulation to depress prefrontal activity during a memory encoding and retrieval task and found bilateral prefrontal recruitment compensating for local inhibition (Davis et al., 2017). However, increased neural activity in dentate/CA3 region and decreased neural activity in anterolateral EC in older adults can lead to pattern separation impairments (Yassa et al., 2011; Reagh et al., 2018). Therefore, the ability to recruit a more distributed neural network with aging must be matched to the task for successful learning.

To support temporal associative learning, there is potential for recruitment of additional connections to compensate for the decreased persistent activity in LEC III. The first part of the compensatory network could come from LEC V, and the second part from the contralateral LEC. Neurons of layer V are also capable of graded persistent firing (Egorov et al., 2002). In an aged brain, reciprocal connections from LEC Vb back to LEC III could provide the additional support for successful memory performance. At this point, how persistent firing in layer V may support learning is still unclear (i.e., does it serve to simply compensate for lower persistent firing ability in layer III, and/or does it convey information about the stimuli). However, its functional connections put it in a prime location to provide compensatory support for LEC III. LEC III also projects contralaterally to the hippocampus and EC (Witter et al., 2017). Bilateral recruitment of the LEC and feedforward projections from LEC Vb together could support temporal associative memory formation, despite a decline in persistent firing ability in LEC III, allowing for cognitive maintenance in aged unimpaired animals.

## How Persistent Firing Is Generated

To understand how aging leads to alterations in persistent firing ability, we must first understand how persistent firing in LEC III is generated.

**FIGURE 7:**
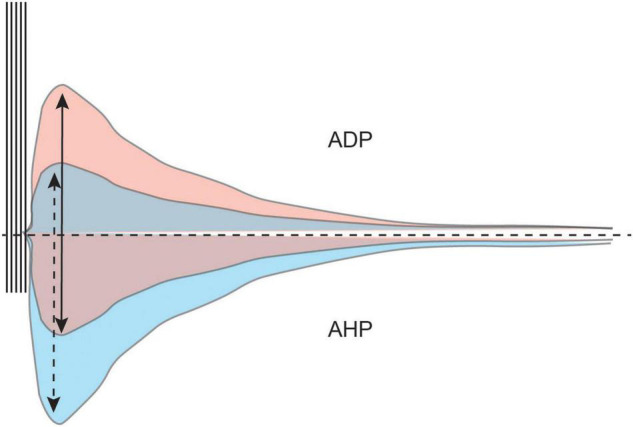
The AHP modulates the size and duration of the ADP. A train of action potentials is typically followed by a hyperpolarization of the membrane potential (AHP, bottom half). The AHP modulates intrinsic excitability and the afterdepolarization (ADP, top half). However, cholinergic activation in the LEC diminishes the AHP and unmasks the ADP. A larger AHP limits the size of the ADP (blue) while a smaller AHP allows for a larger ADP to develop (red).

As we have indicated above, cholinergic modulation facilitates bistability (i.e., stable Up sustained firing and Down quiescent states) in neurons (Hasselmo, 2006; Heys et al., 2012). In a bistable state, incoming stimuli (e.g., suprathreshold direct current injection, or CS information) can easily evoke an Up state. Incoming stimuli will evoke a train of action potentials which will be followed by a depolarization of the membrane potential (afterdepolarization, ADP). The ADP will hold the membrane potential above the threshold for action potentials, and the neuron can continue to fire action potentials even after the removal of external stimulation. Maintenance of the Up state is dependent upon a regenerative plateau potential maintained by continued firing (Figure 7).

For the neuron to achieve bistability, the postburst AHP must first be diminished or completely inhibited. The postburst AHP is the hyperpolarization of the membrane potential that follows a burst of action potentials. It is an important modulator of intrinsic excitability, as the size of the AHP determines the number of action potentials a neuron can fire (Disterhoft and Oh, 2006).

The postburst AHP also modulates the size and duration of the ADP, with a larger AHP limiting the development of the ADP (Wu et al., 2004). For an ADP to be generated that is robust enough to activate an Up state, the AHP needs to be small enough to not suppress the ADP (Figure 7).

Cholinergic activation in the LEC facilitates bistability in the neuron *via* two different pathways. The first is to inhibit the postburst AHP. The second is to activate the channels that underlie the ADP and the plateau potential. Carbachol, the muscarinic agonist used to evoke persistent firing in LEC III neurons *in vitro* is specific to M1 and M3 receptors. M1 and M3 receptors initiate the G_*q*_ second messenger pathway, resulting in the activation of Protein Kinase C (PKC) and inositol triphosphate (IP_3_) (Kubota and Wakamatsu, 2008).

**FIGURE 8:**
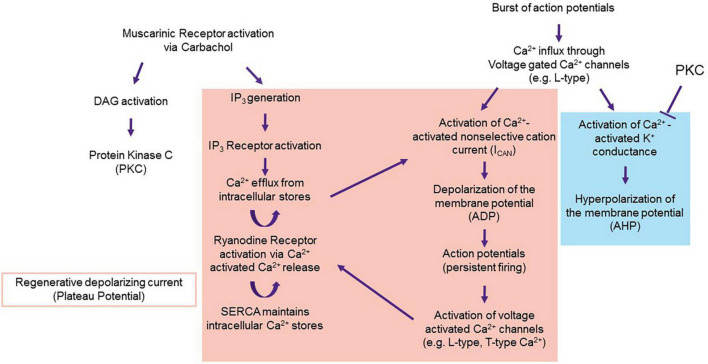
Proposal of the mechanism underlying the ADP. Persistent firing in LEC III starts with muscarinic activation *via* carbachol. Carbachol activates the M1 and M3 pathways, which result in the activation of PKC and IP_3_. PKC inhibits Ca^2+^ activated K+ conductance, disallowing for the generation of the postburst AHP (blue) and unmasking the ADP and the plateau potential (red). Proteins that underlie the ADP and plateau potential may include TRPC (for ADP generation), and voltage gated Ca^2+^ channels and internal calcium stores (for the regenerative plateau potential). From Lin et al. (2020).

As indicated above, the postburst AHP is generated by a burst of action potentials. Action potentials results in the activation of voltage-gated Ca^2+^ channels (predominately L-type) (Deyo et al., 1989; Moyer et al., 1992; Thibault et al., 2001; Gamelli et al., 2011). The Ca^2+^ influx activates a Ca^2+^ activated K+ conductance, which results in a hyperpolarization of the membrane potential (Bond et al., 2004; Disterhoft and Oh, 2006; Hammond et al., 2006; McKay et al., 2012). PKC can block Ca^2+^ activated K+ channels, inhibiting the postburst AHP (Baraban et al., 1985; Disterhoft and Oh, 2006; Tahvildari et al., 2007, 2008; Lin et al., 2020; Figure 8).

Inhibition of the K+ conductance unmasks a cationic influx that depolarizes the membrane potential (i.e., the ADP). Currently, the channels and ionic currents that underlie the ADP and plateau potential are still to be determined. However, it is well-accepted that Ca^2+^ is a necessary component of the ADP. Application of the voltage gated Ca^2+^ channel blocker, Cd^2+^, completely blocks persistent firing (Tahvildari et al., 2008; Lin et al., 2020).

There are many potential sources of Ca^2+^ that underlie the ADP and plateau potential. Stimulation to an LEC III neuron results in a burst of action potentials that could activate voltage gated Ca^2+^ channels, perhaps the same channels as those that eventually lead to the postburst AHP (Fraser and MacVicar, 1996). However, as the K+ channels that underlie the AHP are inhibited by PKC, a depolarization, rather than a hyperpolarization, of the membrane potential follows the initial burst of action potentials.

A body of evidence implicates the Ca^2+^-dependent non-selective cation current (I_*CAN*_) as being a crucial downstream target of the voltage-gated Ca^2+^ conductance. The channel that underlies this current is still unknown. This current may be mediated by its own channel or multiple channels, such as transient receptor potential membrane channels (TRPC) (Tahvildari et al., 2008; Yan et al., 2009; Zhang et al., 2011; Heys et al., 2012). Either way, activation of I_*CAN*_ results in the formation of the ADP. Internal Ca^2+^ stores could contribute to the regenerative plateau potential. M1 and M3 receptor activation of phospholipase C results in increased levels of IP_3_ that would in turn release Ca^2+^ from the endoplasmic reticulum. This, combined with Ca^2+^ from L and T-type channels, could result in a subsequent activation of Ryanodine receptors *via* Ca^2+^ induced Ca^2+^ release (CICR) (Sandler and Barbara, 1999). SERCA pumps located on the ER would restore the Ca^2+^ concentration within the ER. These channels and receptors working in tandem would provide a feedforward mechanism for the regenerative plateau potential that underlies persistent firing.

## Aging-Related Changes Affecting Persistent Firing Ability

With some idea of how persistent firing is generated, we can begin to understand how aging-related changes to the LEC leads to a decrease in persistent firing ability, and subsequent deficits in temporal associative memory. This section will outline a few known aging-related changes to temporal lobe regions. Briefly, this section covers changes to intrinsic excitability, cholinergic innervation, and Ca^2+^ modulation.

### Intrinsic Excitability Changes

A decline in intrinsic excitability within the CA1 region is a well-known consequence of aging, and a primary reason for the cognitive impairments associated with aging. For successful learning, however, increasing intrinsic excitability is both a marker and a prerequisite. An increase in intrinsic excitability is necessary for the successful acquisition of associative memory (Moyer et al., 1996). Decreasing intrinsic excitability impairs acquisition of trace conditioning, while increasing excitability enhances learning of trace conditioning (McKay et al., 2012; Yiu et al., 2014; Volle et al., 2016; Yu et al., 2017).

Studies from our laboratory and others have shown that the intrinsic excitability of CA1 neurons tends to decrease with aging, resulting in the impairments often observed (Power et al., 2002; Disterhoft and Oh, 2006; Matthews et al., 2009). Not all animals, however, exhibit this decrease in excitability as they age. Some subjects retain young-like levels of excitability, and as a result, preserve their learning ability (i.e., aged unimpaired animals) (Moyer et al., 2000; Tombaugh et al., 2005).

Lin et al. (2020) observed a similar relationship between excitability and learning ability in LEC III neurons. Aging caused a decline in excitability, as measured by a larger postburst AHP. While successful acquisition of the trace eyeblink paradigm increased excitability of LEC III neurons in both young and aged animals, aged animals that were unable to acquire the eyeblink paradigm had neurons with significantly larger AHPs compared to young controls and aged unimpaired animals, indicating depressed intrinsic excitability (Lin et al., 2020). The loss of excitability in aged impaired animals is one source of the loss of persistent firing ability in LEC III neurons.

A larger AHP in neurons from aged impaired animals contributes to lower persistent firing ability because the AHP suppresses the ADP. Indeed, Lin et al. (2020) found that along with having a larger postburst AHP compared to young controls and aged unimpaired animals, neurons from aged impaired animals also had a smaller ADP size, accounting for their minimal persistent firing ability (as measured by lower firing probability and slower persistent firing rate). On the other hand, young animals that were trained on tEBC had smaller AHPs compared to young controls and aged impaired animals, and as a result, had larger ADPs, higher firing probability and faster firing rate.

### Degeneration of Cholinergic Innervation in Normal Aging

The basal forebrain regions of the medial septum and vertical and diagonal bands of Broca are highly susceptible to aging-related degeneration. Namely, the regions that supply the cholinergic input to medial temporal regions that are necessary for persistent firing. Several studies have shown the importance of cholinergic innervation in cognition, and especially learning and memory (Winkler et al., 1995; McLin et al., 2002; McKinney and Jacksonville, 2005; Esclassan et al., 2009; Tanninen et al., 2015; Guo et al., 2019). In particular, cholinergic signaling has been shown to be essential in tasks where a neural trace of a stimulus is necessary for successful behavior – i.e., working and temporal associative memory tasks. In one study, application of the muscarinic antagonist scopolamine was shown to impair object and spatial n-back working memory tasks in humans (Green et al., 2005; Hasselmo, 2006). In rats, trace fear and eyeblink conditioning learning were blocked with applications of the M1 receptor antagonist pirenzipine and scopolamine to the EC, respectively (Esclassan et al., 2009; Tanninen et al., 2015).

The basal forebrain demonstrates significant cellular loss in Alzheimer’s and Parkinson’s disease (McKinney and Jacksonville, 2005). In normal aging, these regions are susceptible to dendritic, synaptic, and axonal degeneration, which can contribute to the loss of persistent firing in LEC (Schliebs and Arendt, 2011). There are multiple potential causes for the susceptibility of these neurons to aging-related degeneration, including selective vulnerability to oxidative stress, higher metabolic demand, impairments in gene expression, cytoskeletal transport, and intracellular signaling (Baskerville et al., 2008; Schliebs and Arendt, 2011).

Loss of cholinergic innervation to the LEC would affect persistent firing because neurons in this region would not be able to transition into a bistable state. Lack of IP_3_ Receptor activation means the plateau potential could not be maintained. Loss of cholinergic innervation would also disallow for the elimination of the AHP, which is necessary to achieve bistability.

With the loss of postburst AHP modulation, there is also loss of intrinsic excitability modulation. As mentioned previously, intrinsic excitability within CA1 is a necessary component of learning and is depressed in aging (Disterhoft and Oh, 2006). Within LEC III, Lin et al. (2020) has also shown an increase in excitability following successful learning in both young and aged animals. As a modulator of the postburst AHP, acetylcholine is therefore a modulator of excitability.

Application of cholinesterase inhibitors, such as those used to treat the behavioral symptoms of Alzheimer’s disease, have resulted in an increase in excitability to CA1 neurons. The inhibitors decreased AHP size, resulting in an increase in the number of action potentials fired during a depolarizing current injection. Within aged animals, an amelioration of impairments in acquiring the tEBC task was observed (Kronforst-Collins et al., 1997; Oh et al., 1999; Disterhoft and Oh, 2003; Weible et al., 2004). As a result, loss of cholinergic innervation could have a dual but integrated effect on learning ability in aged animals – the first is the inability of the neuron to achieve bistability, and the second is loss of intrinsic excitability modulation.

### Ca^2+^ Dysregulation in Aging

As indicated above, a fundamental component of both the postburst AHP and the ADP is Ca^2+^. Ca^2+^ underlies many of the channels and currents that are activated for both crucial neuronal mechanisms to occur, including I_*CAN*_, voltage dependent Ca^2+^ channels, and intracellular Ca^2+^ stores. However, Ca^2+^ signaling is altered in normal aging, with “The Calcium Hypothesis” being a well-known theory for the dysfunction that accompanies senescence (Landfield, 1987; Khachaturian, 1989; Thibault et al., 2007; Kumar et al., 2009; Oh et al., 2013). The Calcium Hypothesis posits a dysregulation in Ca^2+^ homeostasis (Ca^2+^ influx, buffering, and extrusion) within the neurons of aged animals. Within the hippocampus CA1, Ca^2+^ dysregulation is thought to underlie the increased postburst AHP and decline in intrinsic excitability within the neurons of aged animals.

One of the sources of altered Ca^2+^ is an increase in L-type conductance within CA1 neurons (Campbell et al., 1996; Thibault and Landfield, 1996; Thibault et al., 2001). As L-type conductance contributes significantly to the postburst AHP, it is one of the major contributors to the decrease in excitability within CA1 neurons (Figure 8). Given that learning and aging-related changes within LEC III neurons reflect that of the CA1 region, it is possible for there to be a similar increase in L-type conductance within LEC III.

Another potential source of Ca^2+^ dysregulation is CICR *via* Ryanodine receptors. Ryanodine receptors promote Ca^2+^ signaling by detecting the presence of Ca^2+^ in the cytosol to release more Ca^2+^ from the ER. Evidence suggests that Ryanodine receptor sensitivity to intracellular Ca^2+^ is enhanced as a result of aging-related oxidative stress (Kumar et al., 2009). CICR is also an important component of the postburst AHP and contributes to the increased AHP that is observed amongst aged animals (Kumar and Foster, 2004; Disterhoft and Oh, 2006; Gant et al., 2015).

However, both L-type channels and CICR also contribute to persistent firing. As a result, it may at first be difficult to reconcile how increased Ca^2+^ could result in a decrease in persistent firing ability. We propose that the linchpin of whether an aged animal maintains its cognitive reserve lies in the postburst AHP. That is, that aging-related Ca^2+^ dysregulation may have a larger effect on the postburst AHP than it does on persistent firing. Since the AHP needs to be eliminated or reduced for bistability to be achieved, an enhanced AHP would make it difficult to achieve and maintain bistability.

## Conclusion

The evidence and theories outlined in this review implicate persistent firing as a vital component of a larger, comprehensive neural system for memory formation. Aging-related alterations to persistent firing in the LEC, either as a result of external (i.e., degeneration of cholinergic innervation) or internal (i.e., loss of intrinsic excitability) influence will result in the impairments that we and others have observed in temporal associative learning.

Future work should seek to first understand how persistent firing in the LEC supports temporal associative learning. In particular, they should investigate whether there are additional functions that persistent firing serves, beyond the canonical theory of supporting the CS during the trace period (e.g., enhancing SC input to CA1).

To develop therapeutics that would alleviate the learning impairments that are so often observed in aging, future work should also seek to examine how the aged unimpaired brain is able to compensate for a decrease in persistent firing ability, allowing for cognitive maintenance. Potential pathways for cognitive maintenance include recruitment of the bilateral LEC III and LEC Vb. Just as importantly, identifying the mechanisms that support persistent firing would allow for the development of therapeutics that could target those channels and proteins so persistent firing ability can be maintained.

## Author’s Note

Sections of this work have been adapted from Lin (2020).

## Author Contributions

CL, MO, and JD conceptualized the work and edited the manuscript. CL drafted the manuscript. All authors contributed to the article and approved the submitted version.

## Conflict of Interest

The authors declare that the research was conducted in the absence of any commercial or financial relationships that could be construed as a potential conflict of interest.

## Publisher’s Note

All claims expressed in this article are solely those of the authors and do not necessarily represent those of their affiliated organizations, or those of the publisher, the editors and the reviewers. Any product that may be evaluated in this article, or claim that may be made by its manufacturer, is not guaranteed or endorsed by the publisher.
